# Fibroblast growth factor 21 and prognosis of patients with cardiovascular disease: A meta-analysis

**DOI:** 10.3389/fendo.2023.1108234

**Published:** 2023-02-28

**Authors:** Bing Yan, Sicong Ma, Chenghui Yan, Yaling Han

**Affiliations:** ^1^ Department of Cardiology, The Second Hospital of Jilin University, Changchun, Jilin, China; ^2^ Department of Cardiology and Cardiovascular Research Institute, Chinese People's Liberation Army, General Hospital of Northern Theater Command, Shenyang, Liaoning, China

**Keywords:** death, prognosis, coronary artery disease, fibroblast growth factor (FGF 21), heart failure, major adverse cardiac event (MACE)

## Abstract

**Background:**

The role of fibroblast growth factor 21 (FGF21) in predicting the long-term prognosis of patients with cardiovascular disease (CVD) remains unknown.

**Methods:**

A comprehensive search in PubMed, Embase, and the Cochrane Library was performed to identify studies reporting the association between FGF21 and prognosis among patients with CVD. A meta-analysis was performed, with patients stratified by coronary artery disease (CAD) or heart failure (HF). The endpoint of CAD or HF was major adverse cardiovascular events defined by each study and a composite of death or HF readmission, respectively. The I^2^ method and linear regression test of funnel plot asymmetry were used to test heterogeneity (I^2^ > 50% indicates substantial heterogeneity) and publication bias (asymmetry P < 0.05, indicating publication bias).

**Results:**

A total of 807 records were retrieved, and nine studies were finally included. Higher FGF21 levels were significantly associated with the risk of major adverse cardiovascular events in patients with CAD (multivariate hazard ratio [HR]: 1.77, 95% confidence interval [CI]: 1.40–2.23, P < 0.05, I^2^ = 0%, fixed-effect model). Increased FGF21 levels were also associated with the risk of all-cause death among patients with CAD (multivariate HR: 2.67, 95% CI: 1.25–5.72, P < 0.05, I^2^ = 64%, random-effect model). No association was found between FGF21 and the endpoint among patients with HF (HR: 1.57, 95% CI: 0.99–2.48, P > 0.05, random-effect model), but a large heterogeneity (I^2^ = 95%) and potential publication bias (Asymmetry P < 0.05) existed in the analysis.

**Conclusion:**

Increased FGF21 levels were independently associated with poor prognosis of CAD, whereas the role of FGF21 in predicting clinical outcomes of HF requires further investigation.

## 1 Introduction

Fibroblast growth factor 21 (FGF21), belonging to the FGF19 subclass of the FGF family, is a pleiotropic endocrine hormone (1) that acts in an autocrine/paracrine manner in multiple tissues (2, 3). FGF21 is induced in white adipose tissue (WAT) by fasting and refeeding and can stimulate glucose entry and increase lipolysis and mitochondrial oxidative capacity (2). FGF21 is a key regulator of energy homeostasis (4), which initiates fat mobilization and increases insulin sensitivity (5–7). Many studies have demonstrated that FGF21 protects against pancreatic damage and β-cell dysfunction and increases glucose transport *via* glucose transporter protein 1 (8). A series of studies have shown that FGF21 has beneficial effects on body weight and glucose and lipid metabolism under physiological conditions (9).

Considering the close association between metabolic syndrome and cardiovascular disease (10), an increasing number of studies have investigated the beneficial effects of FGF21 on the cardiovascular system. Bench et al. reported that FGF21 prevents atherosclerosis (11) and protects against cardiac hypertrophy (12). FGF21 protects against atherosclerosis *via* two independent mechanisms: regulation of adipocyte adiponectin production and suppression of hepatic expression of the transcription factor sterol regulatory element-binding protein-2 (10, 11). Endogenous FGF21 protects against cardiac hypertrophy *via* the sirtuin 1 (SIRT1)–peroxisome proliferator-activated receptor α (PPAR-α) pathway (13). Although a protective role of FGF21 in cardiac function and metabolism has been found, the link between FGF21 and cardiovascular disease is controversial. A series of clinical trials and meta-analyses reported that elevated serum FGF21 levels were associated with an increased incidence of cardiovascular diseases (CVD) (14) as well as cardiovascular mortality among patients with diabetes (15). Collectively, these results from bench research and clinical trials created a paradox in determining the predictive value of FGF21 in CVD. Moreover, most studies have focused on the association between FGF21 levels and the primary prevention of CVD, whereas clinical evidence evaluating the role of FGF21 in the prognosis of patients with CVD is limited. Therefore, we conducted a meta-analysis to explore the association between FGF21 and long-term prognosis of patients with established CVD to provide new evidence unveiling the prognostic role of FGF21 in CVD.

## 2 Materials and methods

### 2.1 Study eligibility and outcomes

The present meta-analysis was performed in accordance with the Preferred Reporting Items for Systematic Review and Meta-analysis Protocols (PRISMA) (16). A comprehensive search was conducted in PubMed, Embase, and the Cochrane Library on June 9, 2022 to identify studies in English that reported the association between FGF21 and the clinical outcomes of patients with CVD. Studies meeting the following inclusion criteria were considered eligible: 1) study population comprising patients with established CVD; 2) studies reporting the relationship between FGF21 levels and CVD prognosis; 3) study endpoints with hard cardiovascular outcomes, such as all-cause or cardiac death, myocardial infarction (MI), and readmission for heart failure (HF); and 4) the follow-up period of studies was at least 6 months from discharge. Using these inclusion criteria and the PICOS (patient, intervention, comparison, outcomes, and study type) principle, we designed the following search terms: “cardiometabolic disease,” “cardiovascular disease,” “coronary artery disease,” “heart failure,” “cardiomyopathy,” “fibroblast growth factor 21,” and “FGF21.” We did not retrieve terms regarding study outcome or type to ensure complete and comprehensive search results (refer to the search strategy in PubMed in the **Supplemental Materials**).

First, the titles and abstracts of the records were reviewed. If relevant, the full texts and references of each record were manually searched and reviewed to evaluate eligibility. No limitations of study type (cohort or case-control study) were included. Conference abstracts were excluded owing to insufficient information. For patients with coronary artery disease (CAD), the primary endpoint was major adverse cardiovascular events (MACE), defined as the composite of ischemic events from each included study, and secondary endpoints included all-cause death and cardiovascular death. For patients with HF, the endpoint was the composite of all-cause death and readmission for HF.

### 2.2 Data extraction and quality evaluation

The following items were extracted from each eligible study: first author, study type, year of publication, patient diagnosis and characteristics, sample size, cutoff value of FGF21 levels, endpoints, follow-up duration, and effect size (event and total numbers, univariate or multivariate hazard ratio [HR]). The authors of the included studies were contacted if key data were unavailable. Observational studies stratified by cohort and case-control studies were evaluated using two versions of the modified Newcastle–Ottawa Scale (NOS) (17, 18). Studies were regarded as high-, medium-, or low-quality if the NOS score was ≥ 7, 5–6, or ≤ 4 points, respectively. All processes of study selection, data extraction, and quality evaluation were performed by two independent reviewers (B. Yan and S. Ma), and discrepancies were finally judged by a third reviewer (C. Yan).

### 2.3 Statistical analyses

The event and total numbers were first calculated as unadjusted risk ratios (RR). Pooled RRs or HRs and 95% confidence intervals (CIs) were synthesized to estimate the impact of FGF21 levels on the observed endpoints using a fixed- or random-effect model if significant heterogeneity existed. Heterogeneity was determined using the Q statistic and I^2^ method and considered significant for P < 0.10 for Q statistic or I^2^ > 50%. Publication bias was detected using funnel plots and a linear regression test for funnel plot asymmetry. Subgroup analysis for the primary endpoint of CAD was conducted among MI sub-populations, and sensitivity analyses were stratified by the effect size (RR derived from event and total numbers or HR) or by omitting each study. A two-sided P < 0.05 was considered statistically significant, except for the heterogeneity test (P < 0.10). All analyses were performed using RStudio (Version 1.2.1335) meta-packages.

## 3 Results

**Figure 1:**
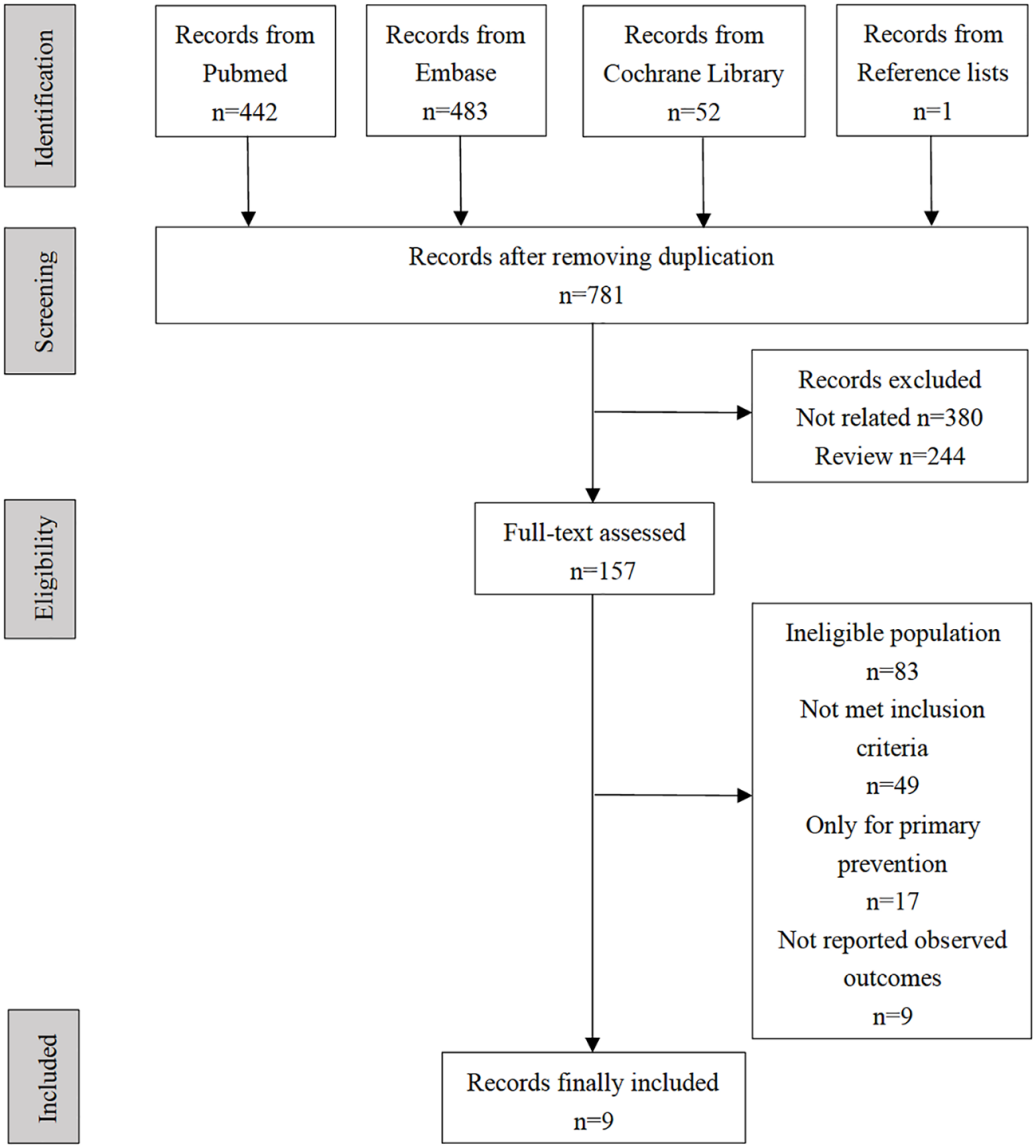
Flowchart of screening eligible studies.

A total of 807 records were identified through a comprehensive retrieval. After removing 27 duplicate studies, titles and abstracts of 781 records were screened. A total of 157 records remained for full-text review, and one record was identified from the reference lists. Finally, nine observational studies that fully met the pre-specified reporting clinical outcomes were included in this meta-analysis (see selection flow diagram in **Figure 1**). During the full-test review, we found that a series of studies reported both FGF21 and cardiovascular prognosis but were finally excluded from our meta-analysis because they were not in accordance with at least one of the inclusion criteria. Representative excluded studies and their respective reasons for exclusion are presented in **Supplemental Materials, Table S1**.

The nine included studies contained five cohort (19–23) and four case-control studies (24–27). Seven studies reported the effect sizes of patients with CAD (19–25), and three studies specifically focused on patients with HF (23, 26, 27). A total of 2674 patients from four studies were included in the analysis of the primary endpoint MACE for CAD (20, 22, 24, 25). A total of 771 patients with HF from three studies were included in the analysis of a composite endpoint of death or readmission for HF (23, 26, 27). Details of the studies included in this meta-analysis are presented in **Table 1**. All included studies were considered medium or high quality with scores ≥ 5 points in the NOS, except for one case-control study (26) that scored 4 points owing to the potential bias of population selection (**Table 2**).

**Table 1:** Characteristics of studies included in the meta-analysis.

Study	Population	Sample size	Study design	Male, n (%)	Age, years ( ± SD)	FGF21 cut-off value (pg/ml)	Endpoints	Follow-up	Effect sizes
**Li 2016**	CAD	1668	Cohort study	1093 (65.5%)	–	Median (IQR): 643.2 (534.7-818.5) *vs* 229.0 (197.4-255.2)	All-cause death and CV death	Median: 4.9 years	Multivariate HR
**Ong 2018**	Stable CAD	1992	Cohort study	–	–	≥281.0 *vs* ≤ 155.0	MACE (CHD death, non-fatal MI, cardiac arrest and stroke)	Median: 4.9 years	Multivariate HR
**Shen 2018**	CAD	169	Case-control study	117 (69.2%)	–	Continuous variable (Logarithm transformation)	MACE (Cardiac death, non-fatal MI, readmission for angina, non-fatal stroke or TVR)	Median: 57 months	Multivariate HR
**Chen 2018**	AMI	165	Case-control study	–	–	≥123.0 *vs* < 123.0	MACE (CV death, recurrent MI, TVR and readmission for HF), all-cause death and CV death	24 months	Multivariate HR for MACE, event/total number for all-cause/CV death
**Gu 2020**	DCM with HFrEF	241	Case-control study	173 (71.8%)	68.8 ± 12.8	≥ 228.4 *vs* < 228.4	A composite of all-cause death and readmission	Mean: 16.1 months	Multivariate HR
**Gan 2020**	CAC	1132	Cohort study	712 (62.9%)	54.2 ± 13.7	≥ 276.1 *vs* < 108.6	SCD	12 months	Event/total number
**Gu 2021**	STEMI with PCI	348	Cohort study	280 (80.5%)	62.1 ± 13.1	≥ 229.8 *vs* < 229.8	MACE (all-cause death and readmission for angina, HF or AMI)	Median: 24 months	Multivariate HR
**Wu 2022**	AHF (including CAD subgroup)	402	Cohort study	234 (58.2%)	70.0 ± 12.0	≥262.0 *vs* < 262.0	All-cause death and a composite of all-cause death and readmission	Median: 193 days	Multivariate HR for all-cause death, event/total number for a composite of all-cause death or readmission
**Fan 2022**	HFrEF	128	Case-control study	86 (67.2%)	70.9 ± 12.4	≥ 231.4 *vs* < 231.4	A composite of all-cause death and HF readmission	Mean: 13.4 months	Univariate HR

SD, standard deviation; FGF21, fibroblast growth factor 21; CAD, coronary artery disease; IQR, interquartile range; CV, cardiovascular; HR, hazard ratio; MACE, major adverse cardiovascular event; CHD, coronary heart disease; MI, myocardial infarction; TVR, target vessel revascularization; AMI, acute myocardial infarction; HF, heart failure; CAC, coronary artery calcification; SCD, sudden cardiac death; ACS, acute coronary syndrome; DCM, dilated HFrEF, heart failure with reduced ejection fraction; STEMI, ST elevation myocardial infarction; PCI, percutaneous coronary intervention; AHF, acute heart failure; OR, odds ratio.

**Table 2:** NOS score of studies included in meta-analysis.

Cohort studies	Selection						Comparability of cohorts based on analysis	Outcome			Total
	Representativeness of exposed cohort		Selection of non-exposed cohort	Ascertainment of exposure		Demonstration that outcome of interest was not present at start of study		Assessment of outcome	Follow-up length	Follow-up adequacy	
**Li 2016**	1		0	1		1	1	1	1	1	7
**Ong 2018**	1		1	1		1	1	1	1	1	8
**Gan 2020**	0		1	1		1	1	0	1	1	6
**Gu 2021**	1		1	0		1	1	1	1	1	7
**Wu 2022**	1		1	1		1	1	1	0	1	7
**Case-control studies**	**Selection**						**Comparability of cases and controls based on analysis**	**Outcome**			**Total**
	**Adequate definition of cases**		**Representativeness of cases**	**Selection of controls**		**Definition of controls**		**Assessment of outcome**	**Follow-up length**	**Follow-up adequacy**	
**Shen 2018**	1		1	1		1	1	1	1	1	8
**Chen 2018**	0		0	1		1	1	1	1	1	6
**Gu 2020**	1		0	0		0	1	0	1	1	4
**Fan 2022**	1		1	0		0	1	0	1	1	5

**Table 2** shows the NOS points of each included studies to evaluate the study quality. Studies were regarded as high quality if scored ≥ 7 points. NOS, Newcastle-Ottawa Scale.

### 3.1 Association between FGF21 and MACE in CAD

**Figure 2:**
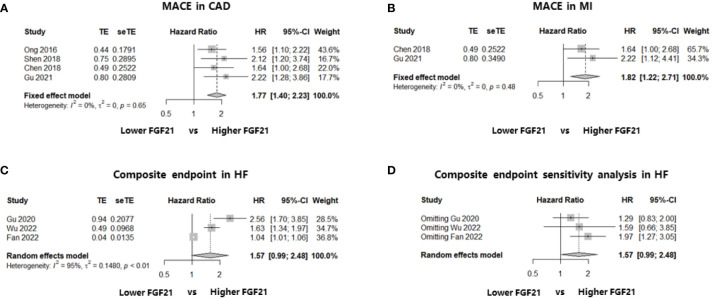
Forest plots of the association of FGF21 with endpoints in patients with CAD or HF. **Figure 2** shows the synthesized effect sizes of FGF21 on predicting endpoints among either CAD or HF patients. The endpoint for CAD and HF was MACE and a composite of all-cause death or HF readmission, respectively. **(A)** FGF21 and MACE in CAD; **(B)** FGF21 and MACE in MI; **(C)** FGF21 and a composite of all-cause death or HF readmission in HF; **(D)** sensitivity analysis of endpoint in HF. FGF21, fibroblast growth factor 21; CAD, coronary artery disease; HF, heart failure; MACE, major adverse cardiovascular event; MI, myocardial infarction.

As mentioned previously, four studies including 2674 patients explored the association between FGF21 levels and long-term MACE among patients with CAD (20, 22, 24, 25). All four studies performed Cox regression analysis and reported multivariate HR as the effect size, and the median follow-up length was at least 24 months (**Table 1**). After effect size synthesis, higher FGF21 levels were independently and significantly associated with the long-term risk of MACE among patients with CAD (multivariate HR: 1.77, 95% CI: 1.40–2.23, P < 0.05, I^2^ = 0%, fixed-effect model; **Figure 2A**). For the subgroup analysis of patients with MI, two studies were included in the analysis (22, 25), and the results showed that higher FGF21 levels were also independently associated with an increased risk of MACE in patients with MI (multivariate HR: 1.82, 95% CI: 1.22–2.71, P < 0.05, I^2^ = 0%, fixed-effect model; **Figure 2B**). To test the stability of the results, sensitivity analysis was performed by omitting each study from the main analysis. The results demonstrated that higher FGF21 levels were consistently and significantly associated with the risk of MACE, irrespective of the removal of any single study (P < 0.05, **Supplemental Materials, Figure S1**). Funnel plots and asymmetry tests of the two analyses showed that no publication bias existed (**Supplemental Materials, Figures S2A, B**).

### 3.2 Association between FGF21 and death in CAD

**Figure 3:**
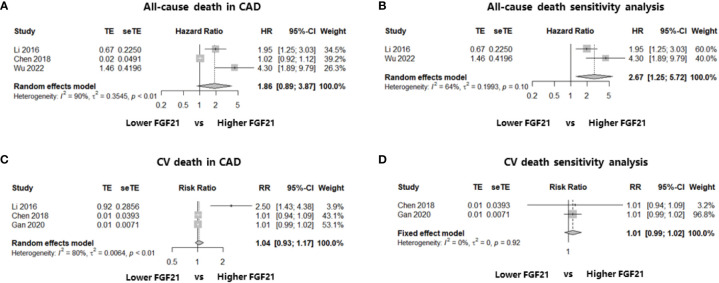
Forest plots of the association of FGF21 with all-cause death or CV death in patients with CAD. **Figure 3** shows the synthesized effect sizes of FGF21 on predicting all-cause death or CV death among CAD patients. **(A)** FGF21 and all-cause death in CAD; **(B)** sensitivity analysis of all-cause death in CAD; **(C)** FGF21 and CV death in CAD; **(D)** sensitivity analysis of CV death in CAD. FGF21, fibroblast growth factor 21; CV death, cardiovascular death.

Three studies including 2235 patients with CAD identified the relationship between FGF21 levels and all-cause death: two cohort studies reporting multivariate HR (19, 23) and one case-control study reporting the event and total numbers (25). We first calculated the RR in this case-control study and then synthesized the RR using multivariate HRs. The results showed that higher FGF21 levels were not associated with the risk of all-cause death among patients with CAD (HR: 1.86, 95% CI: 0.89–3.87, P > 0.05, I^2^ = 90%, random-effect model; **Figure 3A**). However, there was significant heterogeneity (I^2^ = 90%), which may have been because of the mixture of HRs and RR. In addition, funnel plots and asymmetry tests revealed that publication bias may exist (asymmetry P = 0.02; **Supplemental Materials, Figure S3A**). Therefore, we performed a sensitivity analysis by excluding the case-control study without multivariate HR (25), and an independent and significant association between higher FGF21 levels and the risk of all-cause death was found in patients with CAD (HR: 2.67, 95% CI: 1.25–5.72, P < 0.05, I^2^ = 64%, random-effect model; **Figure 3B**).

In terms of FGF21 and CV death, three studies that enrolled patients with CAD were included in the analysis. Two of the three studies reported the event and total numbers (21, 25), and only one cohort study reported the multivariate HR (19). No association was found between FGF21 levels and the risk of CV death among patients with CAD after the effect size synthesis with substantial heterogeneity (RR: 1.04, 95% CI: 0.93–1.17, P > 0.05, I^2^ = 80%, random-effect model; **Figure 3C**). To eliminate the heterogeneity from the mixture of RR and HRs, a sensitivity analysis including the two studies (21, 25) reporting event and total numbers was performed, which also found no significant association between FGF21 and the rate of CV death among patients with CAD (RR: 1.01, 95% CI: 0.99–1.02, P > 0.05, I^2^ = 0%, fixed-effect model; **Figure 3D**). Funnel plots and asymmetry tests found no potential publication bias in the two meta-analyses of CV death (**Supplemental Materials, Figures S3C, D**).

### 3.3 Association between FGF21 and prognosis of HF

Three studies recruiting 771 patients with HF reported the composite endpoint of death or readmission for HF and were included in the meta-analysis (23, 26, 27). The effect sizes of these three studies were event and total numbers (23), univariate HR (27), and multivariate HR (26). The results showed that there was no association between higher FGF21 levels and a composite of death or HF readmission among patients with HF, although there was a statistically significant trend (HR: 1.57, 95% CI: 0.99–2.48, P > 0.05, I^2^ = 95%, random-effect model; **Figure 2C**). We also found a large heterogeneity (I^2^ = 95%) and potential publication bias in this effect size synthesis (asymmetry P < 0.05, funnel plots in **Supplemental Materials, Figure S2C**). A sensitivity analysis, omitting each study, did not significantly change the negative findings (**Figure 2D**).

## 4 Discussion

In this meta-analysis, we explored the association between FGF21 levels and long-term clinical outcomes in patients with CVD stratified by CAD and HF. The results show that higher FGF21 levels were independently associated with the incidence of MACE among patients with CAD. Although the main analysis found no association between FGF21 levels and the rate of all-cause death in CAD, sub-analysis including high-quality studies reporting multivariate HRs showed a significant association between higher FGF21 levels and the risk of all-cause death. In patients with HF, FGF21 was not associated with the rate of a composite of all-cause death or HF readmission, although this outcome should be considered with caution due to the substantial study heterogeneity and variability of effect sizes, including RR, univariate, and multivariate HR. To our knowledge, this is the first meta-analysis to evaluate the association between FGF21 and prognosis of patients with CVD.

FGF21 is a well-known key endocrine hormone that regulates lipolysis in WAT and increases fatty acid oxidation in the liver (28–30). FGF21 increases insulin-independent glucose uptake, improves glucose tolerance, and reduces serum triglyceride levels (31). It has also been recognized that FGF21 has a direct effect on the heart in an endocrine and autocrine manner, which is mediated by the FGFR and co-receptor β-Klotho (32). FGFR1 and FGFR3 are the main FGF21 receptors in the heart (33, 34). FGF21 binds to FGFR and the co-receptor β-Klotho in cardiomyocytes and activates downstream signaling pathways, including ERK, AMPK, and the SIRT1-PPAR-α pathway (35, 36). Among these receptors, FGF21 exerts cardioprotective effects, mainly through FGFR1 (37). A recent study also showed that sodium/glucose cotransporter-2 inhibitors (SGLT2i) can increase serum FGF21 levels, which is one of the mechanisms underlying the cardioprotective effects of SGLT2i (38). Several preclinical trials have also demonstrated that mimics and long-acting derivatives of FGF21 have beneficial effects on body weight, lipoprotein profiles, and metabolic homeostasis (39, 40). However, previous clinical trials have reported that elevated FGF21 levels are associated with increased cardiovascular risk and mortality (41–45). Obviously, a paradox between basic research and clinical studies exists regarding the definite role of FGF21 and CVD; therefore, further comprehensive studies are needed to resolve this issue.

One of the main findings of this meta-analysis was that high FGF21 levels were independently and significantly associated with an increased long-term risk of MACE in patients with CAD (multivariate HR: 1.77, 95% CI: 1.40–2.23, P < 0.05, I^2^ = 0%, fixed-effect model). Even when focusing on the MI subgroup, the result was consistent (multivariate HR: 1.82, 95% CI: 1.22–2.71, P < 0.05, I^2^ = 0%, fixed-effect model). No study heterogeneity or publication bias was found in the statistical analyses, indicating that these results were stable and reliable. In terms of all-cause death and FGF21 among patients with CAD, the meta-analysis did not find a significant association (HR: 1.86, 95% CI: 0.89–3.87, P > 0.05, I^2^ = 90%, random-effect model). However, high study heterogeneity from a mixture of multivariate HRs and RR may discount credibility. Therefore, a sensitivity analysis including studies reporting multivariate HRs was conducted, and an independent and significant association was found between higher FGF21 levels and the risk of all-cause death in CAD. Similar meta-analyses were also performed to determine the relationship between FGF21 and CV death in patients with CAD, but no significant associations were found, irrespective of the main outcome, including three studies (RR: 1.04, 95% CI: 0.93–1.17, P > 0.05, I^2^ = 80%, random-effect model) or the sensitivity analysis including two studies reporting RR (RR: 1.01, 95% CI: 0.99–1.02, P > 0.05, I^2^ = 0%, fixed-effect model). Nevertheless, we should note that the study sample size involved in CV death was small; more importantly, two of the three studies only reported event and total numbers without adjusting for other multiple factors, which is inferior to the multivariate HR for authentically reflecting the effect size. Therefore, elevated FGF21 levels are independently associated with poor long-term prognosis in patients with CAD, although more high-level evidence is warranted.

For patients with HF, we pre-specified a composite of all-cause death and HF readmission as endpoints. After statistical analysis, there was no significant association between FGF21 and the long-term endpoint of patients with HF (HR: 1.57, 95% CI: 0.99–2.48, P > 0.05, I^2^ = 95%, random-effect model). The sensitivity analysis showed that the result was positive only when a case-control study that reported a univariate HR was excluded. The study heterogeneity was also high owing to the effect size variability reported by the included studies (including RR, univariate HR, and multivariate HR). Hence, although a negative relationship was found between FGF21 and clinical outcomes in patients with HF, this result may be insufficient to determine the definite relationship between FGF21 and the prognosis of patients with HF and needs to be reconfirmed by more clinical trials. Overall, our meta-analysis and previous findings (14, 15, 45) collectively identify that increased FGF21 levels may be an independent predictor of poor prognosis among patients with CVD, rather than a protective factor of the heart, which was found in mechanistic studies. The FGF21 paradox not only exists in primary prevention but also in the long-term prognosis of CVD. This paradox may be because of a compensatory response to metabolic stress in patients with CVD. FGF21 resistance may be another underlying mechanism, according to recent findings reporting that stress conditions can decrease FGF21 co-receptor β-Klotho expression in the heart, impair FGF21 signaling, and weaken the protective effect of FGF21 on cardiomyocytes (46). Both underlying mechanism studies and high-level clinical trials are needed to determine this uncertainty to provide potential drug targets.

The present meta-analysis had several limitations. First, although comprehensive retrieval was performed and nine studies were finally included, the study sample size was also relatively small. Second, the effect sizes reported by the included studies were varied and uneven, including event/total number, odds ratio, and univariate and multivariate HR. This diversity increases the study heterogeneity, which may influence data synthesis. Moreover, as shown in **Table 1**, the cutoff values of FGF21 used in the included studies varied without a uniform criterion. Therefore, a definite FGF21 cutoff value for predicting cardiovascular risk still needs to be explored. Finally, the study endpoints were not abundant because of limited data obtained from the included studies. These deficiencies require further clinical trials to fill the gap.

This meta-analysis demonstrated that increased FGF21 levels were independently associated with the long-term prognosis of patients with CAD. In patients with HF, no association was found between FGF21 levels and prognosis, and the role of FGF21 in predicting clinical outcomes remains unclear. The FGF21 paradox exists in the long-term prognosis of CVD.

## Data availability statement

The original contributions presented in the study are included in the article/**Supplementary Material**. Further inquiries can be directed to the corresponding authors.

## Author contributions

BY designed this study. SM wrote the main manuscript and prepared figures. CY conducted the manuscript reviewing and editing. YH supervised all these procedures of this study. All authors contributed to the article and approved the submitted version.
