# Supplementary material for: Fibroblast growth factor 21 and prognosis of patients with cardiovascular disease: A meta-analysis
Source: Front Endocrinol (Lausanne). 2023 Feb 28;14:1108234. doi: 10.3389/fendo.2023.1108234 (PMC10011636; doi:10.3389/fendo.2023.1108234)
Supplement: Supplementary file 1 [file DataSheet_1.docx]

Supplemental materials

**Fibroblast Growth Factor 21 and Prognosis of Patients with Cardiovascular Disease during Secondary Prevention: A Meta-analysis**

Bing Yan MD, Sicong Ma MD, Chenghui Yan MD, PhD*, Yaling Han MD, PhD*

*** Correspondence:**Yaling Han, MD, PhD, Department of Cardiology, General Hospital of Northern Theater Command, Shenyang, Liaoning, 110016, China, e-mail: [hanyaling@163.net](mailto:hanyaling@163.net).

Chenghui Yan, MD, PhD, Department of Cardiology, General Hospital of Northern Theater Command, Shenyang, Liaoning, 110016, China, e-mail: [yanch1029@163.com](mailto:yanch1029@163.com)

**Search strategy in Pubmed**

((("cardiometabolic"[All Fields] OR "cardiometabolically"[All Fields]) AND ("disease"[MeSH Terms] OR "disease"[All Fields] OR "diseases"[All Fields] OR "disease s"[All Fields] OR "diseased"[All Fields])) OR ("cardiovascular diseases"[MeSH Terms] OR ("cardiovascular"[All Fields] AND "diseases"[All Fields]) OR "cardiovascular diseases"[All Fields] OR ("cardiovascular"[All Fields] AND "disease"[All Fields]) OR "cardiovascular disease"[All Fields]) OR ("coronary artery disease"[MeSH Terms] OR ("coronary"[All Fields] AND "artery"[All Fields] AND "disease"[All Fields]) OR "coronary artery disease"[All Fields]) OR ("heart failure"[MeSH Terms] OR ("heart"[All Fields] AND "failure"[All Fields]) OR "heart failure"[All Fields]) OR ("cardiomyopathie"[All Fields] OR "cardiomyopathies"[MeSH Terms] OR "cardiomyopathies"[All Fields] OR "cardiomyopathy"[All Fields])) AND ("fibroblast growth factor 21"[Supplementary Concept] OR "fibroblast growth factor 21"[All Fields] OR ("fibroblast growth factor 21"[Supplementary Concept] OR "fibroblast growth factor 21"[All Fields] OR "fgf21"[All Fields])

**Table S1. Representative excluded studies and respective reasons for exclusion**

| **Study** | **Population** | **Sample size** | **Study design** | **FGF21 cut-off value (pg/ml)** | **Endpoints** | **Follow-up** | **Effect sizes** | **Reasons for exclusion** |
| --- | --- | --- | --- | --- | --- | --- | --- | --- |
| Zhang 2015(1) | **AMI and non-AMI control** | 100 | Case-control study | Continuous variable (Logarithm transformation) | All-cause death, MI and readmission | **30 days** | Multivariate OR | **Ineligible population including non-AMI control, and the short follow-up period was insufficient to observe long-term prognosis** |
| Chou 2016(2) | **HFpEF and healthy control** | 238 | Case-control study | ≥ median vs < median | All-cause death, readmission for HF | 1 year | Event/total number | **Ineligible population including healthy control** |
| Shen 2017(3) | **CAD and NAFLD** | 218 | Cohort study | Continuous variable (Logarithm transformation) | Cardiac death | Median: 5 years | Multivariate HR | **The population was replicated with one already included study(4), and also included both CAD and NAFLD** |
| Wong 2018(5) | CAD | 1166 | Cohort study | **NA** | MACE (ACS, PAD, stroke and CV death) | Median: 35 months | Multivariate HR | **The negative HR of FGF21 was not shown in the manuscript** |
| Xie 2022(6) | MI after CABG | 265 | Cohort study | Continuous variable | MACE (re-infarction, stroke, HF and death) | **48 hours** | Multivariate OR | **The short follow-up period was insufficient to observe the long-term prognosis** |

FGF21: fibroblast growth factor 21; AMI: acute myocardial infarction; MI: myocardial infarction; OR: odds ratio; HFpEF: heart failure preserved ejection fraction; HF: heart failure; CAD: coronary artery disease; NAFLD: non-alcoholic fatty liver disease; HR: hazard ratio; NA: not available; MACE: major adverse cardiovascular events; ACS: acute coronary syndrome; PAD: peripheral artery disease; CV: cardiovascular; CABG: coronary artery bypass grafting.

**Figure S1. Sensitivity analysis of MACEs in CAD patients**


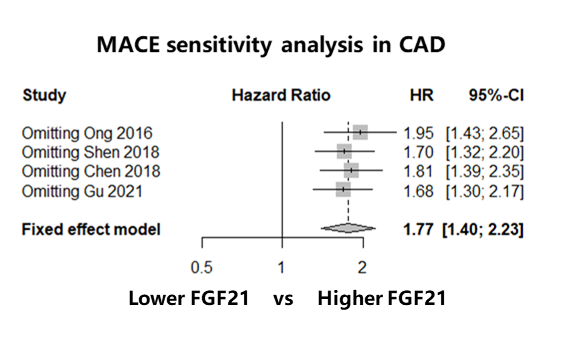


Figure S1 shows the sensitivity analysis of FGF21 on predicting MACE among CAD patients by omitting each one included study. The results were consistent with the main outcome, irrespective of omitting any one study. MACE: major adverse cardiovascular event; CAD: coronary artery disease; FGF21: fibroblast growth factor 21.

**
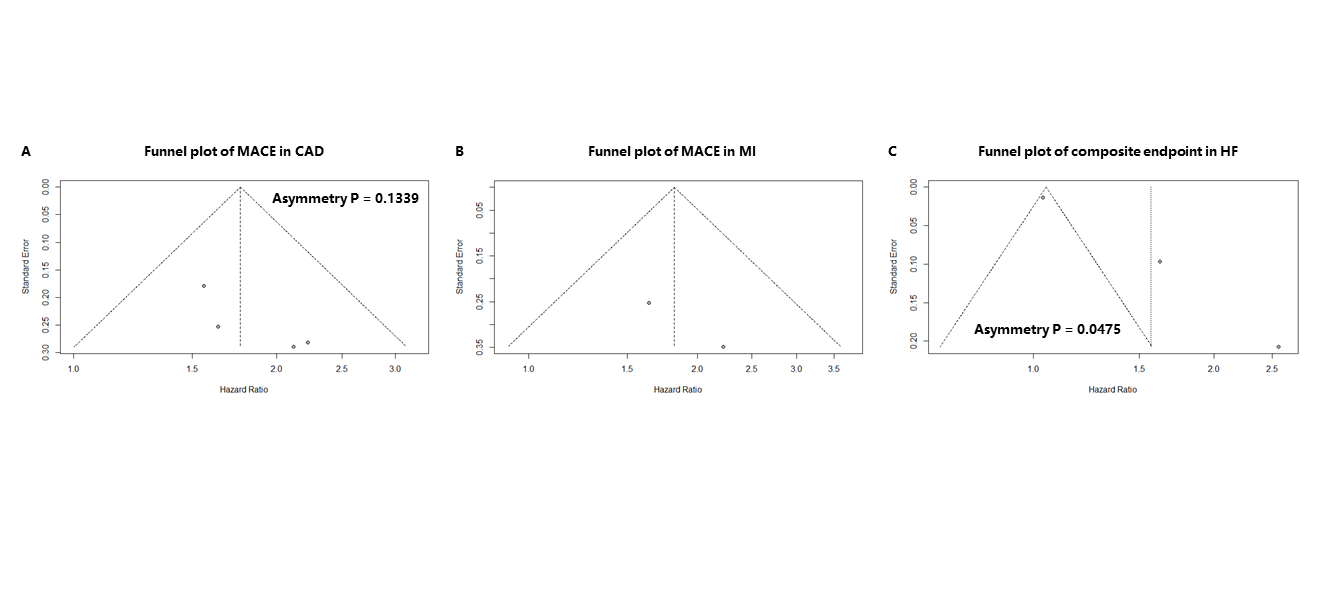
Figure S2. Funnel plots of meta-analyses of MACEs in CAD and the Composite endpoint in HF**

Figure S2 shows the funnel plots and asymmetry tests for publication bias of meta-analyses of MACEs among CAD and the composite of death or HF readmission among patients with HF. P-value was calculated by linear regression test of funnel plot asymmetry. A) Funnel plot of MACE in CAD patients; B) Funnel plot of MACE in patients with MI; C) Funnel plot of a composite of death or HF readmission in patients with HF. When included only 2 studies in the analysis, linear regression test of funnel plot asymmetry was unable to conduct due to the small sample size (Figure S2B). MACE: major adverse cardiovascular event; CAD: coronary artery disease; HF: heart failure; MI: myocardial infarction.

**Figure S3. Funnel plots of meta-analyses of all-cause death or CV death in CAD patients
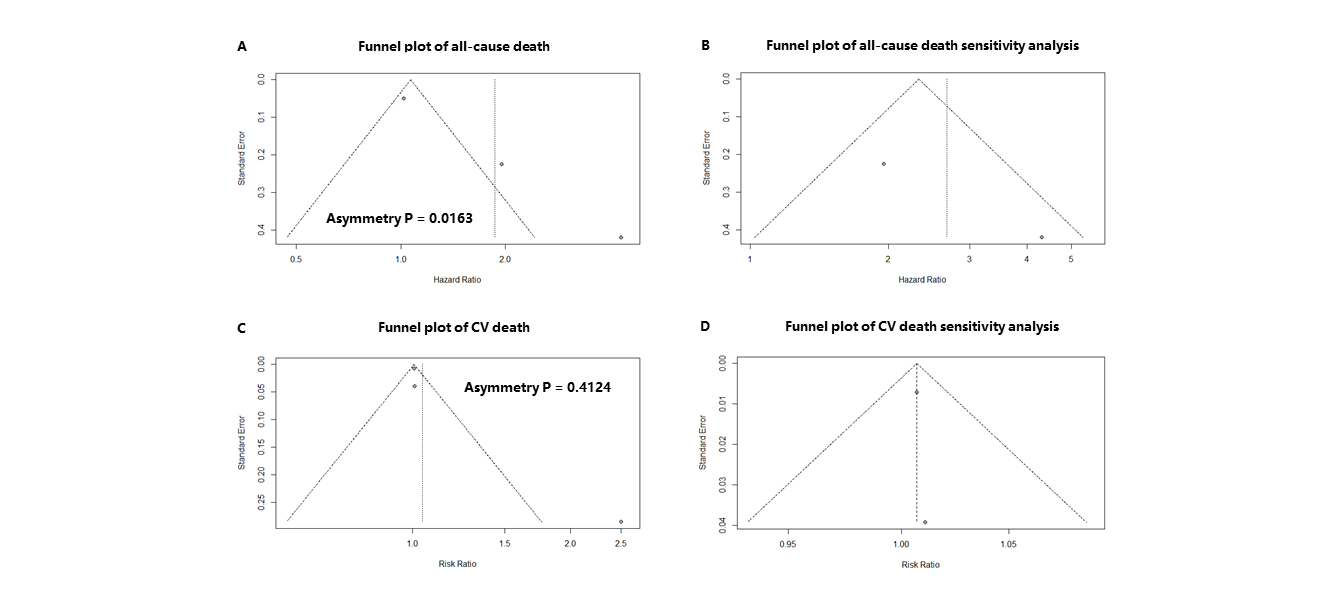
**

Figure S3 shows the funnel plots and asymmetry tests for publication bias of meta-analyses of all-cause death or CV death among CAD patients. P-value was calculated by linear regression test of funnel plot asymmetry. Funnel plot of: A) All-cause death; B) All-cause death sensitivity analysis; C) CV death; D) CV death sensitivity analysis. When included only 2 studies in the analysis, linear regression test of funnel plot asymmetry was unable to conduct due to the small sample size (Figure S3B and S3D). CV death: cardiovascular death; CAD: coronary artery disease.

**REFERNCES**

1. Zhang W, Chu S, Ding W, Wang F. Serum Level of Fibroblast Growth Factor 21 Is Independently Associated with Acute Myocardial Infarction. PLoS One. 2015;10(6):e0129791.

2. Chou RH, Huang PH, Hsu CY, Chang CC, Leu HB, Huang CC, et al. Circulating Fibroblast Growth Factor 21 is Associated with Diastolic Dysfunction in Heart Failure Patients with Preserved Ejection Fraction. Sci Rep. 2016;6:33953.

3. Shen Y, Zhang X, Pan X, Xu Y, Xiong Q, Lu Z, et al. Contribution of serum FGF21 level to the identification of left ventricular systolic dysfunction and cardiac death. Cardiovasc Diabetol. 2017;16(1):106.

4. Shen Y, Zhang X, Xu Y, Xiong Q, Lu Z, Ma X, et al. Serum FGF21 Is Associated with Future Cardiovascular Events in Patients with Coronary Artery Disease. Cardiology. 2018;139(4):212-8.

5. Wong YK, Cheung CYY, Tang CS, Au KW, Hai JSH, Lee CH, et al. Age-Biomarkers-Clinical Risk Factors for Prediction of Cardiovascular Events in Patients With Coronary Artery Disease. Arterioscler Thromb Vasc Biol. 2018;38(10):2519-27.

6. Xie W, Li D, Shi Y, Yu N, Yan Y, Zhang Y, et al. Serum FGF21 Levels Predict the MACE in Patients With Myocardial Infarction After Coronary Artery Bypass Graft Surgery. Front Cardiovasc Med. 2022;9:850517.
